# Stabilization of Deformable Nanovesicles Based on Insulin-Phospholipid Complex by Freeze-Drying

**DOI:** 10.3390/pharmaceutics11100539

**Published:** 2019-10-16

**Authors:** You Xu, Yiyue Guo, Yuqi Yang, Yingying Meng, Xuejun Xia, Yuling Liu

**Affiliations:** State Key Laboratory of Bioactive Substance and Function of Natural Medicines, Beijing Key Laboratory of Drug Delivery Technology and Novel Formulations, Department of Pharmaceutics, Institute of Materia Medica, Chinese Academy of Medical Sciences & Peking Union Medical College, Beijing 100050, China; youxupumc@gmail.com (Y.X.); guoyiyue@imm.ac.cn (Y.G.); yangyuqi@imm.ac.cn (Y.Y.); mengyingying@imm.ac.cn (Y.M.); ylliu@imm.ac.cn (Y.L.)

**Keywords:** deformable nanovesicles, combination, cryoprotectant, mechanism, insulin

## Abstract

Deformable nanovesicles have been extensively investigated due to their excellent ability to penetrate biological barriers. However, suffering from serious physical and chemical instabilities, the wide use of deformable nanovesicles in medical applications is still limited. Moreover, far less work has been done to pursue the lyophilization of deformable nanovesicles. Here, we aimed to obtain stable deformable nanovesicles via freeze-drying technology and to uncover the underlying protection mechanisms. Firstly, the density of nanovesicles before freeze-drying, the effect of different kinds of cryoprotectants, and the types of different reconstituted solvents after lyophilization were investigated in detail to obtain stable deformable nanovesicles based on insulin-phospholipid complex (IPC-DNVs). To further investigate the underlying protection mechanisms, we performed a variety of analyses. We found that deformable nanovesicles at a low density containing 8% lactose and trehalose in a ratio of 1:4 (8%-L-T) have a spherical shape, smooth surface morphology in the lyophilized state, a whorl-like structure, high entrapment efficiency, and deformability after reconstitution. Importantly, the integrity of IPC, as well as the secondary structure of insulin, were well protected. Accelerated stability studies demonstrated that 8%-L-T remained highly stable during storage for 6 months at 25 °C. Based on in vivo results, lyophilized IPC-DNVs retained their bioactivity and had good efficacy. Given the convenience of preparation and long term stability, the use of combined cryoprotectants in a proper ratio to protect stable nanovesicles indicates strong potential for industrial production.

## 1. Introduction

Since the discovery by Bangham in 1965, liposomal delivery technologies have been extensively investigated for delivering a wide variety of medicinal agents. Among these technologies, novel lipid vesicles known as deformable or elastic (flexible) vesicles, are particularly attractive due to great ability to penetrate the stratum corneum (SC) barrier and deliver loaded drugs into the epidermis and dermis layers, and even attain systemic circulation [1,2]. Representative deformable nanovesicles include transfersomes [3], ethosomes [4], invasomes [5], spanlastics [6], glycerosomes [7], penetration enhancer-containing vesicles (PEVs) [8], hyalurosomes [9], ultradeformable nanovesicles [10] and transethosomes [11]. In general, the deformability of such vesicles is achieved by an edge activator that is capable of destabilizing the lipid bilayers of the vesicles [12]. Like traditional liposome, deformable nanovesicles suffer serious physical and chemical instability during long term storage [13], which limit their widespread use in medical applications. After long-term storage, the risks are high that deformable nanovesicles suffer from leakage of the encapsulated drug and particle size alters due to the instability of the lipid bilayers [14,15]. Vesicle aggregation, hydrolysis, and oxidation of the phospholipid may also have unwanted effects during long-term storage [16,17]. So far, no product has been introduced on the market. Therefore, the preparation of stable deformable nanovesicles is necessary to translate them into marketable products.

Many studies have monitored the stability of deformable nanovesicles. However, their results indicated that nanovesicles suffered severe drug leakage and changes in particle size during the storage, which normally occurred at 4 °C and lasted for 2 to 6 months [6,10,15,18]. Although some promising results were obtained, the monitoring time was usually too short to meet the requirements for industrial production [19,20]. Stability studies on biomacromolecule loaded deformable nanovesicles are rare. Recently, new strategies, such as increasing the solution stability and storing the samples at freezing temperatures, were used to stabilize deformable nanovesicles, achieving good results [21,22,23]. Nevertheless, achieving uniform specifications in samples prepared using these strategies is difficult, and the conditions required for transportation and storage are harsh. The freeze-drying technology (lyophilization) has been proven to be an effective method to prevent the hydrolysis of phospholipids and the physical degradation of conventional vesicles [24,25,26,27]. When a product is in solid form, interfacial stress due to agitation during transport and handling can be easily eliminated. Therefore, lyophilized samples are easy to transport and store. However, far less work has been conducted to lyophilized deformable nanovesicles. Lyophilizing deformable nanovesicles presents a challenge due to the instability of these structures. Thus, a novel lyophilization strategy to prepare stable deformable nanovesicles is highly desirable.

Here, we aimed to achieve the long-term stability of deformable nanovesicles. Previous studies showed that although deformable nanovesicles based on the insulin-phospholipid complex (IPC-DNVs) have an excellent in vivo hypoglycemic effect and also had no visible mucosal irritation to the buccal mucosa. However, they were not stable after 15 days of storage and were prone to insulin precipitation, increasing particle size and decreasing drug entrapment efficiency (EE, %). We systematically investigated the effect of different nanovesicles density before lyophilization, and reconstituted solvent after lyophilization. Also, different kinds of cryoprotectants were examined for the stability of IPC-DNVs. Firstly, we evaluated these effects via the appearance, particle size, deformability index (DI), EE (%) and long-term stability of lyophilized samples. Different ratios and amounts of combined cryoprotectants were evaluated for better protection. Secondly, differential scanning calorimetry (DSC) was used to detect the glass transition temperature (T_g_) of lyophilized deformable nanovesicles, transmission electron microscopy (TEM) was used to investigate the microstructure of nanovesicles before freeze-drying and after reconstitution, and scanning electron microscopy (SEM) was used to explore the surface morphology of deformable nanovesicles after freeze-drying. X-ray diffraction (XRD) and circular dichroism (CD) were conducted to assess the effect of freeze-drying on the integrity of IPC and the secondary structure of the insulin, respectively. Moreover, the accelerated stability studies were used for the prediction of long-term storage stability. Finally, an in vivo study was used to confirm the relative bioavailability (*F_r_*) and pharmacological relative bioavailability (*F_p_*) of the lyophilized samples after reconstitution.

## 2. Materials and Methods 

### 2.1. Materials

Recombinant human insulin was purchased from Dongbao Enterprise Group Co., Ltd. (Tonghua, Jilin, China). Lecithin (70% phosphatidylcholine/30% phosphatidyl-ethanolamine, lipoid phospholipid) was obtained from Shanghai Toshisun Biology and Technology Co., Ltd. (Shanghai, China). Sodium deoxycholate and D-(+)-trehalose dehydrate purchased from Sigma (St. Louis, MO, USA), α-lactose monohydrate purchased from Aladdin (Shanghai, China). Other chemicals and solvents were of analytical or chromatography grade.

### 2.2. Animals

Male big-ear Japanese rabbits were purchased from Beijing Huafukang Bioscience Co., Inc. (Beijing, China), and were raised at the Institute of Material Medical, Chinese Academy of Medical Sciences and Peking Union Medical College (Beijing, China). The experiments were performed with the approval of the Laboratory Animal Care and Use Committee of Peking Union Medical College on 10 April 2017 and the animal experiment lasted to 28 December 2017 (project identification code 00005780). All animal experiments were performed under the laboratory animal guidelines for ethical review of animal welfare (GB_T 35892-2018) for the welfare of the animals (China).

### 2.3. Preparation of Stable Deformable Nanovesicles

#### 2.3.1. Preparation of Nanovesicles

IPC-DNVs were prepared using a thin-film hydration method, as described in our previous studies [28,29]. Briefly, 60 mg insulin and 600 mg phospholipid were independently dissolved in 6mL 0.1% trifluoroacetic acid–methanol and 60 mL dichloromethane, respectively. After mixing for 10 minutes, the organic solvents were removed to form an insulin-phospholipid complex. Then, 660 mg complex, 600 mg phospholipid and 400 mg Tween 20 were dissolved in 20mL dichloromethane to form a clear solution. The solvent was evaporated to form a beehive film. The film was hydrated with sodium deoxycholate (SDCs, 100 mg) and phosphate-buffered saline (PBS, pH 7.4, 18.4 mL). Finally, the suspension was sonicated to form smaller vesicles. The final concentration of insulin in the nanovesicles was approximately 87 IU/mL.

#### 2.3.2. Lyophilization

**Figure 1:**
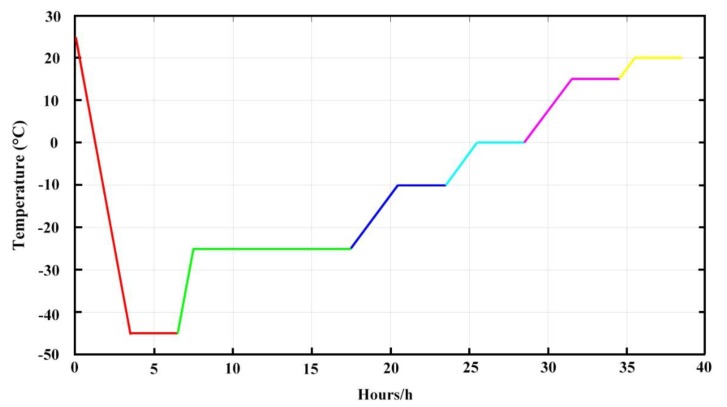
Freeze-drying processes.

A certain amount of IPC-DNVs were diluted with PBS into 1 mL, transferred to a 10 mL glass vial (Gerresheimer Group GmbH, Düsseldorf, Germany), covered with a special stopper (Hualan Co., Ltd, Jiangyin, Jiangsu, China) for lyophilization and placed in an Epsilon 1-4 LSCplus freeze-dryer (Martin Chris, Osterode am Harz, Germany). For freezing the samples, the sample plate was cooled at 20 °C/h from 20 to −45 °C under atmospheric pressure (according to the DSC result of different cryoprotectant solvents where the highest glass transition temperature (*T*_g’_) was −28 °C, and the freezing temperature and should be 10 to 15 °C lower than *T*_g’_, so we chose −45 °C as the pre-freeze temperature). After 3 h, the pressure was reduced to 0.05 mbar, and the plate was heated (5 °C /h) to −25 °C (according to the DSC result of different cryoprotectant solvents where the lowest eutectic point (T_e_) was −16.23 °C, and the drying temperature should be 5 to 12 °C lower than *T*_e_, so we chose −25 °C as the main drying temperature). The samples were dried under these conditions for 10 h. Afterward, the plate was slowly heated (5 °C /h) to −10 °C for 3 h, heated (5 °C /h) to 0 °C for 3 h, and then heated (5 °C /h) to 10 °C for 3 h, finally, the plate was left for 3 h for the sample to return to room temperature (Figure 1). The lyophilized deformable nanovesicles were then flushed with nitrogen gas and the vials were closed directly inside the freeze dryer.

To study the effect of different densities of deformable nanovesicles, we lyophilized IPC-DNVs without dilution. Simultaneously, 0.5, 0.33, or 0.2 mL of freshly prepared IPC-DNVs were diluted with PBS into 1 mL. The dilution multiples were as follows: 0, 1, 3, 5. To investigate the impact of different kinds of cryoprotectant on the properties of lyophilized deformable nanovesicles, 8% cryoprotectant was dissolved in deformable nanovesicles immediately after preparation. Finally, different ratios and amounts of cryoprotectants were used according to the experimental design.

#### 2.3.3. Reconstitution

The lyophilized samples were reconstituted by dispersion with medium to the original volume by manual shaking for 10 s at room temperature.

### 2.4. Determination of Particle Size, Polydispersity Index(PDI) and Density

The average particle size, PDI of IPC-DNVs and the reconstituted IPC-DNVs were determined using a particle size analyzer (Malvern Zetasizer Nano ZS at an angle of 173°, WR, UK) at 25 ± 0.5 °C. All experiments were repeated three times.

The density of deformable nanovesicles was calculated according to the following equation:

The total number of nanovesicles N can be obtained as:

(1) $$N=\frac{\left(m_{1}/\rho_{1}+m_{2}/\rho_{2}\right)}{\left(\frac{4\pi }{3}\times \left(R_{1}{}^{3}-R_{2}{}^{3}\right)\right)}\;,$$

where *m*_1_ refers to the amount of phospholipid, *ρ*_1_ refers to the density of phospholipid, *m*_2_ refers to the amount of Tween 20, *ρ*_2_ refers to the density of Tween 20, *R*_1_ is the radius of the outer layer (particle size according to the TEM result), *R*_2_ is the radius of the inner layer (according to the TEM result), and $\left(\frac{4\pi }{3}\times \left(R_{1}{}^{3}-R_{2}{}^{3}\right)\right)$ is the average volume of every nanovesicle.

Then, the number of nanovesicles per unit volume α, which is the density of the nanovesicles, can be described as:

(2) $$\alpha =\frac{N}{V}\;,$$

where V is the volume of solution.

### 2.5. Drug Content and Entrapment Efficiency (EE, %)

The concentration of insulin was determined using reverse-phase high-performance liquid chromatography (RP-HPLC) with an Agilent Technologies 1200 series HPLC system (Agilent, Santa Clara, CA, USA) and a 300SB-C18 column (4.6 × 250 mm, 5 μm, Agilent, Palo Alto, CA, USA). RP-HPLC were used under 0.2 M sulfate buffer/acetonitrile (74:26, *v*/*v*), 1 mL/min flow rate, 214 nm ultraviolet detection, 20 μL injection volume, and 40 °C column oven temperature.

The EE (%) of the nanovesicles was determined by using a fast ultrafiltration method. Briefly, the amount of insulin in 1 mL nanovesicle was labeled with *W_total-insulin._* Then, 1 mL nanovesicles was placed in the upper of a centrifugal filter tube (Amicon Ultra-4 centrifugal devices, 100K NMWL, Millipore, Billerica, MA, USA) and centrifuged at 4,000 rpm for 40 min to separate the free and entrapped insulin. After ultrafiltration, the filtered nanovesicles in the lower of the centrifugal filter tube was collected (*W_free-1_*), the unfiltered nanovesicles in the upper of the centrifugal filter tube was discarded and washed with PBS in one time. Then, the upper of the centrifugal filter tube was washed three times with PBS at 2500 rpm for 10 min to collect the insulin in the lower of the centrifugal filter tube (*W_free-2_*). Then, the amount of insulin in the ultrafiltrates (*W_free-insulin=_ W_free-1_* + *W_free-2_*) was measured by RP-HPLC. The EE was calculated according to the following equation:

(3) $$EE=\left(\frac{w_{total-insulin}-w_{free-insulin}}{w_{total-insulin}}\right)\times 100\%,$$

where *W_total- insulin_* is the total amount of insulin in nanovesicles, *W_free-insulin_* is the sum of the amount of insulin in the ultrafiltrates. All experiments were repeated three times.

### 2.6. Deformability Index of Nanovesicles

The deformability of nanovesicles was determined using a stainless-steel pressure filter device [30]. Deformable nanovesicles were extruded through polycarbonate membranes with a pore diameter of 50 nm (*r_p_*) at a constant pressure of 0.45 MPa for 5 min. The filtered deformable nanovesicles were collected. After extrusion, the particle size of the deformable nanovesicles (*r_v_*) was measured using a particle size analyzer mentioned in Section 2.4. The deformability index (DI, g/cm^2^/s) of the deformable nanovesicles was calculated according to the following equation:

(4) $$DI=J*{\left(\frac{r_{v}}{r_{p}}\right)}^{2},$$

where *J* is the rate of penetration through the permeability membrane, *r_v_* is the particle size after extrusion, and *r_p_* is the pore diameter of the permeability membrane (50 nm). All experiments were repeated three times.

### 2.7. X-Ray Diffraction (XRD)

XRD measurements were performed using a Rigaku D/max-2550 diffractometer (Rigaku Instrument, Tokyo, Japan). Lyophilized deformable nanovesicles were subjected to CuKα radiation under 40 kV and 150 mA over the 2θ range from 3° to 80° at a rate of 8°/min in 0.02° increments.

### 2.8. Differential Scanning Calorimetry (DSC)

DSC measurements were performed using a Pyris-1 calorimeter (Perkin-Elmer, Foster City, CA, USA). Approximately 10 mg of lyophilized samples were accurately weighed and placed into standard aluminum pans. A reference scan of the empty pan was subtracted. The samples were cooled to −50 °C with a scan rate of 5 °C /min and maintained stable for 1 min, then heated to 80 °C with a scan rate of 5 °C /min.

### 2.9. Microscopic Examination of Nanovesicles

#### 2.9.1. Scanning Electron Microscopy (SEM)

The lyophilized samples were fixed on the aluminum stubs and coated with gold for 480 s in an auto fine coater (JFC-1600, JEOL, Tokyo, Japan), the morphologies and microstructure of the lyophilized samples were characterized by a field emission FESEM (S8010, Hitachi, Tokyo, Japan), and an accelerating voltage of 15 kV was used.

#### 2.9.2. Transmission Electron Microscopy (TEM)

The microstructure of the reconstituted nanovesicles was explored using a TEM-1400plus (JEOL, Tokyo, Japan) at 120 kV. After diluting 50 times with 5% glycerol solution, the sample was deposited on a carbon support membrane (Zhongjing Keyi Technology Co., Ltd., Shanghai, China) and was allowed to stand for 5 min. Then the excess liquid was absorbed using filter paper. Finally, the sample was negatively stained by adding a drop of 1% phosphotungstic acid, allowed to stand for 5 minutes, and dried at 25 °C.

### 2.10. Conformational Stability

The integrity of the secondary structure of insulin after releasing from nanovesicles was verified by CD studies. The released insulin was obtained using the fast ultrafiltration method. Briefly, after treating with an appropriate amount of ethanol, 1 mL of the prepared nanovesicle was placed in a centrifugal filter tube and centrifuged at 4000 rpm for 40 min to obtain released insulin. CD spectra were acquired at 25 °C using a spectropolarimeter (J-815 Spectropolarimeter, Jasco, Tokyo, Japan) in the far ultraviolet region. The sample was placed in a 1-mm pathlength cell and scanned at a rate of 50 nm/min. The lamp housing was purged with nitrogen. The insulin concentration was 15 μg/mL (diluted with PBS). A reference scan of the PBS was subtracted. All experiments were repeated three times.

### 2.11. Stability Study

The lyophilized IPC-DNVs were stored at 25 °C for 6 months. The appearance of the lyophilized samples, the insulin content, the particle size, PDI, entrapment efficiency, deformability index and micro-structure of deformable nanovesicles were determined after 0, 1, 2, 3 and 6 months. All experiments were repeated three times.

### 2.12. Moisture Content Determination

The moisture content of the lyophilized deformable nanovesicles was measured via a Karl Fischer titration instrument (905 Titrando, Metrohm, Switzerland). After the instrument reached equilibrium, lyophilized deformable nanovesicles were accurately weighed and placed inside the titration chamber. The lyophilized sample was dissolved at 100 rpm. The moisture content was recorded from the instrument. All experiments were repeated three times.

### 2.13. In Vivo Study

#### 2.13.1. Determination of Serum Insulin and Relative Insulin Bioavailability of Lyophilized IPC-DNVs

The relative bioavailability of insulin delivered by the buccal administration of lyophilized IPC-DNVs was evaluated in normal rabbits. Food was withheld 2 h prior to the experiment but they were allowed free access to water. The rabbits were anesthetized with injection of Nembutal (2%, 50 mg/kg) from a marginal ear vein. Then, the esophagus of each rabbit was surgically ligated to prevent rabbits from swallowing. For the buccal administration, the mouth cavity was cleaned by a cotton ball. The drug was divided into four parts, two parts for the sublingual, and two parts for left and right buccal mucosa. The esophagus was untied 30 min after administration. The entire experiment was conducted out under anesthesia, and the Nembutal was supplemented every 1 h (2%, 10 mg/kg).

The rabbits were randomly divided into groups of three each: Insulin-SC and the lyophilized IPC-DNVs-SC which received solution by subcutaneous injection (1 IU/kg), IPC-DNVs and the lyophilized IPC-DNVs groups were administered through the mucosa (10 IU/kg). Blood samples were collected from the ear veins at 0, 0.5, 1, 1.5, 2, 2.5, 3, 4, 5, and 6 h after administration, and the blood serum was separated by centrifugation at 3500 rpm for 10 min at 4 °C. Serum insulin content was quantified using an insulin ELISA kit (Mercodia AB, Uppsala, Sweden).

The relative bioavailability (Fr, %) of the lyophilized IPC-DNVs after buccal administration was calculated according to the following equation:

(5) $$F_{r}=\frac{AUC_{buccal}\times Dose_{s.c.}}{AUC_{s.c.}\times Dose_{buccal}}\times 100\%,$$

where *Dose_buccal_* and *Dose_s.c._* are the insulin doses administered buccally or subcutaneously, respectively, and *AUC* values are the area under the curve of serum insulin content over time.

#### 2.13.2. Bioactivity and In Vivo Hypoglycemic Effect of Lyophilized IPC-DNVs

The relative pharmacological bioavailability of the lyophilized IPC-DNVs was evaluated in normal rabbits. The preparation of rabbits was the same as that used in Section 2.13.1.

The rabbits were randomly divided into groups of three each: the insulin group that received insulin via the mucosa as control, the Insulin- SC and the lyophilized IPC-DNVs-SC which received solution by subcutaneous injection (1 IU/kg), and IPC-DNVs group and the lyophilized IPC-DNVs group were administered through the mucosa (10 IU/kg). Blood samples were collected from the rabbit ear veins at 30-min intervals from 0 to 6 h after administration. The blood glucose levels were measured using OneTouch Ultra (Johnson & Johnson, New Brunswick, NJ, USA). The relative pharmacological bioavailability (*Fp*) of insulin after buccal administration was calculated according to the following equation:

(6) $$F_{p=}\frac{AAU_{buccal}\times Dose_{s.c.}}{AAC_{s.c.}\times Dose_{buccal}}\times 100\%,$$

where *Dose_buccal_* and *Dose_s.c_*_._ are the insulin doses administered buccally or subcutaneously, and *AAC* values are the area above the curve of reduction in blood glucose level over time.

### 2.14. Statistical Analysis

The data are expressed as the mean ± standard deviation (SD) of three independent experiments. Statistically significant differences between groups were determined by student’s t-test using SPSS software (SPSS Inc., Chicago, IL, USA). p-value of less than 0.05 was considered statistically significant: *p < 0.05, **p < 0.01, and ***p < 0.001.

## 3. Results

### 3.1. Formulation Development and Corresponding Protection Mechanism Consideration

#### 3.1.1. Effect of Different Density of Nanovesicles and Reconstituted Solvent

**Figure 2:**
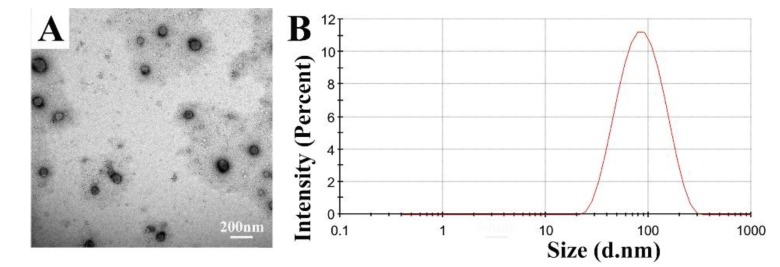
(**A**) Morphology and (**B**) size distribution of insulin-phospholipid complex deformable nanovesicles (IPC-DNVs).

As described above, IPC-DNVs were stable for 15 days. The particle sizes were 82.55 ± 3.85 nm with a narrow size distribution indicated by PDI < 0.200. The entrapment efficiency and deformability index of IPC-DNVs were 79.29% ± 2.41% and 38.25 ± 1.38 g/cm^2^/s (*n* = 3) before lyophilization, respectively [29]. TEM micrographs of IPC-DNVs confirmed their spherical shape and whorl-like structure (Figure 2A). Figure 2B shows IPC-DNVs have a narrow particle size distribution.

Table 1 shows the trend in size, PDI, entrapment efficiency, and deformability index of lyophilized IPC-DNVs with different density of nanovesicles. When IPC-DNVs were lyophilized without dilution, nanovesicles had a much larger size and higher PDI which indicated particle aggregation and instability. Appropriate dilution can effectively prevent aggregation during the freeze-drying process which can be observed from their decreased size. However, when the dilution multiple was 5 times, entrapment efficiency decreased significantly, indicating insulin was leakage and deformable nanovesicles were instability. For a relatively smaller size, lower PDI and higher entrapment efficiency, the dilution multiple was selected to be 1 or 3 times dilution. However, the dilution multiple should not be too high, otherwise the volume of the same sample will be very large. Under such circumstances, the drying time needs to be extended, which increases the cost of production. The required container will be much larger, which is unsuitable for industrial production. Therefore, we chose a 1-time dilution.

Table 2 shows the effect of the different reconstituted solvents on the features of lyophilized IPC-DNVs. Different reconstituted solvents had little effect on the deformability index of the nanovesicles. When reconstituted with the ionic solution, the particle size was higher than that of water. Among them, acetate buffer and sodium chloride had influenced the encapsulation of insulin. Therefore, water was selected as the reconstituted solvent.

#### 3.1.2. Effect of Single Cryoprotectant

Cryoprotectants, such as commonly used carbohydrates, glycine, and some new cryoprotectants, such as hyaluronan [31], were evaluated. The effects of using different cryoprotectants on IPC-DNVs are shown in Table 3. The insulin contents in all reconstituted samples preserved with cryoprotectants were in the range of 90% to 110%, except in samples preserved with mannitol, which was lower than 90% due to drug leakage. Sample appearance, size, PDI, entrapment efficiency, and deformability index were calculated to evaluate the effect of the cryoprotectants. Compared with the stability of IPC-DNVs, the stability of all samples improved. Comparatively, in the presence of lactose or trehalose, both lyophilized and thawed forms demonstrated superior performance. For trehalose, reconstituted samples had a relatively low particle size with a narrow distribution, and high entrapment efficiency and deformability index at initial, whereas particle size and deformability index changed slightly after 1 month of storage. Besides, the PDI of trehalose increased, indicating the nanovesicles had aggregated to some extent. For lactose, the particle size and entrapment efficiency of the reconstituted samples changed considerably. However, we observed almost no change in particle size, PDI, entrapment efficiency, and deformability index after 1 month of storage. Given the advantages and disadvantages of the trehalose and lactose, we chose trehalose and lactose as a mixed cryoprotectant and further investigated the impact of their ratios and amount.

#### 3.1.3. Effect of Using Combinations of Cryoprotectants

The results obtained using different ratios of lactose and trehalose are shown in Table 4. For a different ratio, the content of cryoprotectants is fixed at 8%. When lactose and trehalose were used at ratios of 1:4 to 1:8, the lyophilized deformable nanovesicles had a good appearance, small particle size, low PDI, good entrapment efficiency, and deformability index after freeze-drying. However, after storage for 2 months, samples prepared at ratios of 1:4–1:6 maintained their particle size, PDI, entrapment efficiency, and deformability index. According to result of the effect of single cryoprotectant, lactose had good ability in maintaining the particle size and deformability index in the long term. Hence, we selected 1:4 for further investigation.

**Figure 3:**
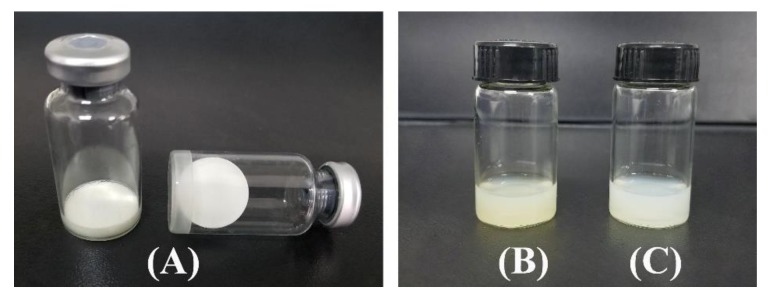
Appearance of lyophilized 8%-L-T, as well as the appearance of reconstituted 8%-L-T. (**A**) lyophilized IPC-DNVs (8%-L-T), (**B**) IPC-DNVs, (**C**) reconstituted 8%-L-T.

The results obtained using different amounts of cryoprotectants are shown in Table 4. For different amounts, lyophilized IPC-DNVs using lactose and trehalose in a ratio of 1:4 as cryoprotectants. In the range of 5% to 13%, the lyophilized samples had a good appearance in both the lyophilized and reconstituted states. When the amount of cryprotectants was 5% to 8%, the samples had a relatively small particle size, narrow size distribution, and good entrapment efficiency and deformability index after freeze-drying and storage for 1 month. Comparatively, samples prepared with 8% cryoprotectant better maintained their size and distribution after 2 months of storage at 25 °C. For a longer storage period, 8% cryoprotectant was found to be the optimal concentration. Samples prepared with 8% lactose and trehalose had a caked and compact appearance after freeze-drying. The surface of samples were also very smooth (Figure 3A). The 8% cryoprotectant also had an excellent appearance after reconstitution that was the same as that of IPC-DNVs (Figure 3B, C). Therefore, 8% lactose and trehalose in a ratio of 1:4 (8%-L-T) were determined to be the optimal cryoprotectant formulation. To further investigate the underlying protection mechanism of 8%-L-T, the experiments in the following sections were performed with 8% lactose (8%-L) and 8% trehalose (8%-T) as references.

#### 3.1.4. Differential Scanning Calorimetry

A higher glass transition temperature (*T*_g_) is essential for cryoprotectants to prevent membrane fusion and drug leakage [32]. The *T*_g_ of the lyophilized sample is depicted in Table 5. Trehalose had a high *T*_g_. However, the T_g_ of lactose was relatively low. With the combination of trehalose and lactose (8%-L-T), the *T*_g_ of the system increased, which is important for long-term storage of the product.

#### 3.1.5. Microscopic Examination of Nanovesicles

**Figure 4:**
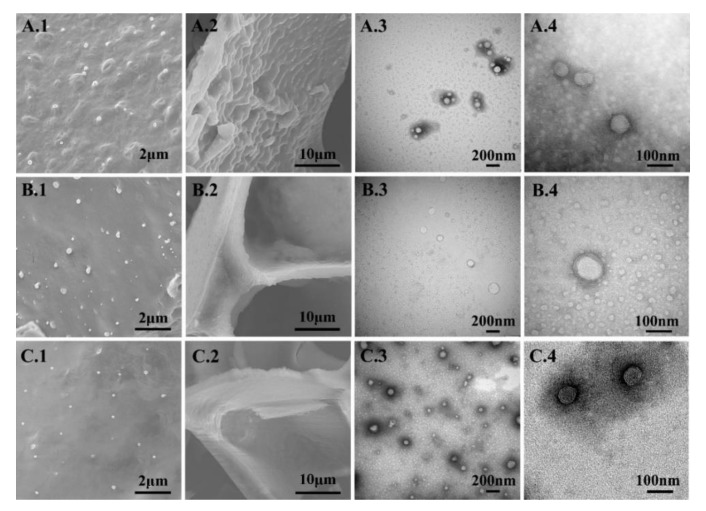
Micrographs of lyophilized and reconstituted samples. (**A**) 8%-T, (**B**) 8%-L, and (**C**) 8%-L-T, **1** and **2** are SEM images, **1** morphology at low magnification and **2** morphology at high magnification, **3** and **4** are TEM images, **3** micro-structure at low magnification and **4** refers to micro-structure at high magnification.

SEM studies were performed on the lyophilized nanovesicles to elucidate the effects of lactose, trehalose, and their combination on the morphology of nanovesicles and their support structure (Figure 4). TEM studies were used to observe the microstructure of nanovesicles after reconstitution (Figure 4). 

Figure 4 shows the different nanovesicle structures when protected with 8%-T, 8%-L, and 8%-L-T. For 8%-T (Figure 4 A1, A2), the surface of the support structure was rough with many salient peaks, whereas the size of the lyophilized nanovesicles was well controlled. After reconstitution (Figure 4 A3, A4), the particle size was well controlled, but some nanovesicles aggregated, and the whorls were fuzzy. For 8%-L (Figure 4 B1, B2), the samples formed porous structures that were smooth and rigid, and the nanovesicles did not directly contact each other. However, the particle size increased, and the whorls of deformable nanovesicles were completely invisible after reconstitution (Figure 4 B3, B4). The morphology of the nanovesicles was less discrete, spherical and smooth, the shell of samples prepared with 8%-L was thinner than before. When combined, samples prepared with a mixture of the two cryoprotectants (Figure 4 C1, C2) contained nanovesicles that were spherical with a smooth surface. The support structure of 8%-L-T was as strong as that of 8%-L and much smoother than that of 8%-T. The final lyophilized samples were caked and compact, and they could reconstitute in 10 s with only a slightly altered particle size. We observed obvious whorl-like structures of 8%-L-T, which were the same as the IPC-DNVs before lyophilization (Figure 4 C3, C4).

#### 3.1.6. X-Ray Diffraction

**Figure 5:**
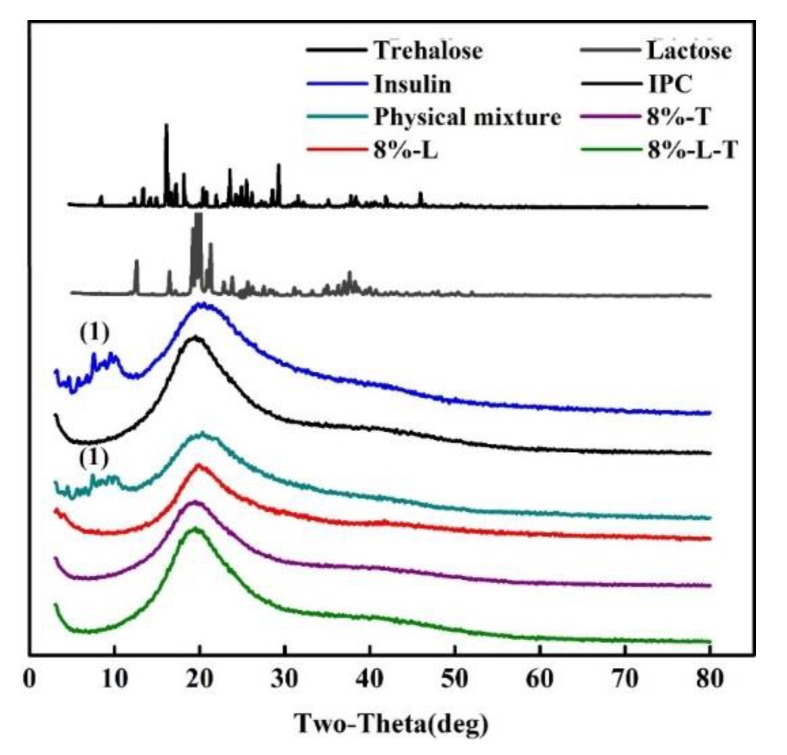
X-ray diffraction patterns (1 refers to diffraction peaks of insulin).

The XRD patterns of insulin, IPC, physical mixture (a mixture of insulin and phospholipid in a ratio of 1:20), and lyophilized nanovesicles are shown in Figure 5. A marked crystalline form can be seen from lactose and trehalose. However, no crystals appeared in the lyophilized samples and they were in amorphous form. The XRD patterns of insulin and the physical mixture exhibited numerous diffraction peaks (1), which were indicative of insulin’s diffraction peaks. In contrast, no characteristic peaks of insulin were observed in the IPC and lyophilized samples, indicating that the integrity of complex was not disrupted during freeze-drying process.

#### 3.1.7. Conformational Stability

**Figure 6:**
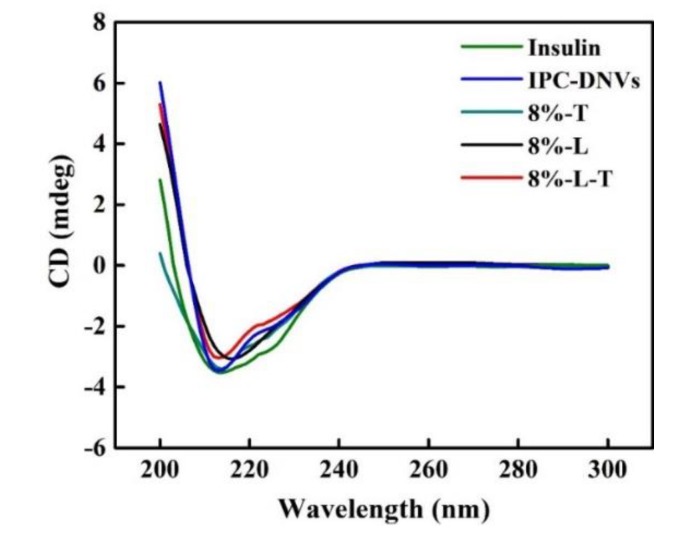
CD spectra of samples before and after lyophilization.

Figure 6 shows the CD spectra of insulin before lyophilization and after reconstitution. Table 6 shows the α-helix and θ208/θ223 ratios of lyophilized IPC-DNVs. The ratio of θ208/θ223 is applied to measure the quantity of insulin association [33]. When two monomers dimerized, an antiparallel β-structure will be formed, resulting in an increase in θ223 without an increase in θ208.

The CD spectra showed insulin, IPC-DNVs, 8%-L-T, and 8%-T have two minima at 208 and 223 nm, which is a typical α-helix structure. This is in close agreement with the spectra obtained by others [33,34]. However, the CD spectra of 8%-L were smooth and had no minima at 208 and 222 nm. For 8%-L, the α-helix ratio of insulin increased to 35.9%, which indicated that the secondary structure of insulin had changed, providing a negative result. The θ208/θ223 ratio of 8%-L was 2.30, indicating that the structure of insulin had tightened. For 8%-T, the α-helix ratio of insulin was 24.1%, and the θ208/θ223 ratio was 1.42. The θ208/θ223 ratio of insulin in 8%-L-T was 1.61, which is similar to that of insulin in solution. The lyophilized state of deformable nanovesicles can appropriately maintain the secondary structure of insulin.

#### 3.1.8. Stability Studies

**Figure 7:**
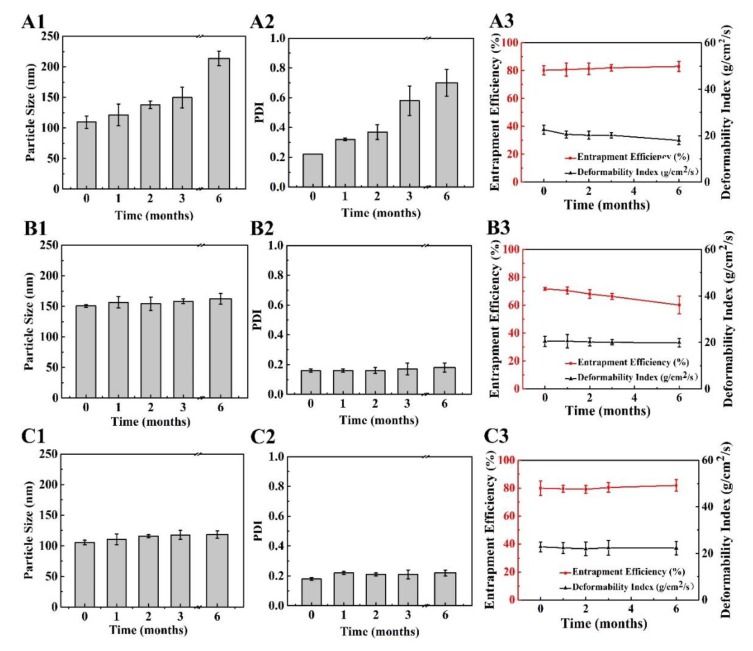
Changes in lyophilized deformable nanovesicles after storage at 25 °C for a period of 6 months. (**A**) 8%-T, (**B**) 8%-L, and (**C**) 8%-L-T, **1** particle size, **2** PDI, **3** entrapment efficiency (EE) and deformability index (DI).

**Figure 8:**
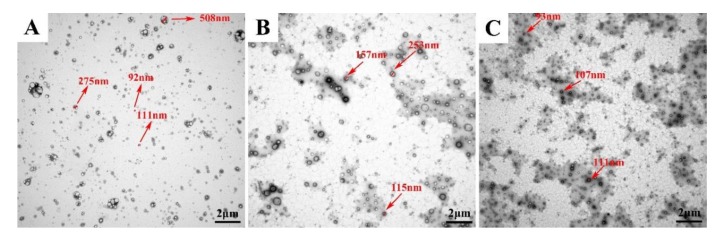
Changes in lyophilized deformable nanovesicles after storage at 25 °C for a period of 6 months. (**A**) 8%-T, (**B**) 8%-L, and (**C**) 8%-L-T.

Based on the results in previous sections, the stability of, 8%-T, 8%-L, and 8%-L-T were examined during 6 months of storage at 25 °C. After long-term storage, the lyophilized samples still maintained their cake-like shape and compact appearance. As shown in Figure 7A and Figure 8A, when protected by 8%-T, most of the particles aggregated during long-term storage, resulting in larger particle size and PDI, and a lower deformability index than initially. However, 8%-T showed a stable entrapment efficiency which meant insulin has no leakage during storage. For 8%-L (Figure 7B), the particle size, PDI and entrapment efficiency were stable during long-term storage. However, their deformability index decreased, and all of the particles appeared relatively large in TEM images (Figure 8B). When lactose and trehalose were combined as a cryoprotectant, no significant changes in size, PDI, entrapment efficiency, or deformability index were observed over the storage period of 6 months (Figure 7C). The particles were consistent in size and had no contact with each other (Figure 8C). The content of insulin for all samples was maintained in the range of 90% to 110% during the 6 months storage period. Therefore, 8%-L-T appeared to be a promising cryoprotectant for preparing stable deformable nanovesicles.

#### 3.1.9. Moisture Content Determination

Low moisture content is an essential criterion for any lyophilized products. According to regulatory authorities, the maximum acceptable water content in lyophilized samples is within 3% (*w*/*w*) [35]. The lyophilized deformable nanovesicles were found to have a low moisture content of 1.82% ± 0.18% after stored at 25 °C for 6 months, which is within the allowable limit.

### 3.2. Bioavailability and In Vivo Hypoglycaemic Effect of Lyophilized IPC-DNVs

**Figure 9:**
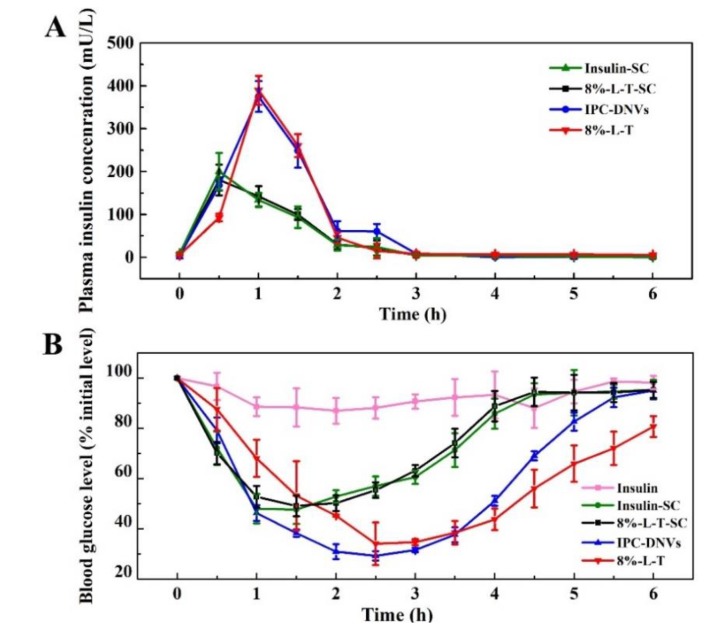
Time-dependent (**A**) plasma insulin concentrations (**B**) and in vivo hypoglycaemic effect.

Figure 9A shows the plasma insulin concentration after subcutaneous administration of insulin (insulin-SC) and 8%-L-T (8%-L-T-SC) and buccal administration of IPC-DNVs and 8%-L-T. To identify the bioavailability of lyophilized deformable nanovesicles, 8%-L-T-SC was subcutaneously administrated. 8%-L-T-SC had the same hypoglycemic properties as insulin-SC, which meant insulin could be quickly released from the nanovesicles. IPC-DNVs and 8%-L-T have the same pharmacokinetics characteristics. The peak plasma (serum) concentrations of IPC-DNVs and 8%-L-T were attained at 1 h and had the same height. Table 7 summarizes the AUC and *Fr* of the four groups. Compared with the subcutaneous administration of insulin solution, the Fr of IPC-DNVs and 8%-L-T were 18.27% and 17.45%, respectively. The freeze-drying process appeared to have no effect on the bioavailability of 8%-L-T.

The hypoglycemic effects on lyophilized deformable nanovesicles are shown in Figure 9B. The freeze-drying process had little effect on the biological activity of 8%-L-T-SC (96.61%) compared with insulin-SC. After buccal administration, the glucose levels of animals treated with insulin solution barely changed within 6 h, whereas the hypoglycemic effect was evident in those treated with IPC-DNVs and 8%-L-T. When compared with IPC-DNVs, 8%-L-T had the same trend in the hypoglycemic effect (Table 7). The maximum decrease in blood glucose was approximately 65%, whereas 8%-L-T caused a slightly prolonged glucose reduction compared with IPC-DNVs. Thus, the in vivo hypoglycemic effect indicates that using 8% lactose and trehalose in a ratio of 1:4 as a mixed cryoprotectants (8%-L-T) can considerably protect the integrity of deformable nanovesicles.

## 4. Discussion

In this study, we found that among commonly and currently used cryoprotectants, trehalose and lactose have a much better protective effect on deformable nanovesicles. When lactose and trehalose were used in combination, IPC-DNVs had constant size, PDI, entrapment efficiency, deformability index, insulin content, microstructure, excellent hypoglycaemic effect after freeze-drying and had good long-term stability.

From the observation of the effects of different density of nanovesicles and reconstituted solvents, we found that different density of nanovesicles directly determined the number of nanovesicles in per unit volume. In this study, we used a simple formula to calculate the density of nanovesicles. The optimal density of nanovesicles should be such that there is no direct contact among individual nanovesicles. The density of nanovesicles before dilution is relatively high, almost in direct contact with each other (1000 nanovesicle per μm^3^). Therefore, appropriate dilution can effectively prevent aggregation during the freeze-drying process. Different reconstituted solvents, considerably influence the reconstituted process of deformable nanovesicles. The addition of ions to micro-particles usually has a demulsification effect, so the addition of ions might disrupt the integrity of deformable nanovesicles [36]. In order to explore the effect of ions on the reconstitution of deformable nanovesicles, we included the zeta potential of reconstituted nanovesicles. However, it showed that the reconstituted deformable nanovesicles displayed high negative potential (−25 to −40 mV, determined using a particle size analyzer (Malvern Zetasizer Nano ZS at an angle of 173°) at 25 ± 0.5 °C) which meant the reconstituted solvents had no influence on zeta potential (IPC-DNVs, −26.2 ± 0.5 mV).

The protection of the microstructure is crucial as it directly determines the deformability of deformable nanovesicles. In this study, SDCs and Tween 20 were used as edge activators to destabilize the lipid bilayers of the nanovesicles so that the nanovesicles could be highly deformable. When edge activators were inserted into the phospholipid bilayers, a larger distance between the phospholipid molecules was produced and the order of phosphatide acyl was disrupted [37]. Under this situation, monosaccharides like glucose are not suitable to act as croyprotectants during storage, as the *T*_g_ (<40 °C) of monosaccharides are so low that the microstructures of deformable nanovesicles tend to collapse. Some rigid cryoprotectants, such as some oligosaccharides (Inulin), are not suitable for the lyophilization of deformable nanovesicles since they are struggling to accommodate the irregular surface of deformable nanovesicles. For polysaccharides, despite their higher *T*_g_, most of their lyophilized samples are viscous and their lyophilized structures are dense or difficult to reconstitute. This multi-hydroxyl in one polysaccharides (hyaluronan) cause their deformable nanovesicles to aggregate easily or disrupt nanovesicles when being reconstituted. The large size of polysaccharides limits the flexibility of chains and causes steric hindrance, so achieving a compact coating at the surface of deformable nanovesicles is difficult. For cryoprotectants, the ability to protect the micro-structure of deformable nanovesicles depends on molecular flexibility. Therefore, among the various kinds of cryoprotectants, choosing disaccharides for deformable nanovesicles is preferable.

From our characterization of lyophilized IPC-DNVs, we found lactose and trehalose have different protection mechanisms. For 8%-T, the presence of the trehalose helped IPC-DNVs form smaller nanovesicles during the freeze-drying process, as reported previously [38]. Trehalose also helped to maintain low molecular mobility of individual nanovesicles, and reduced freezing and dehydration stress during freeze-drying. Trehalose could protect against dehydration stress between the inside and outside of nanovesicles by accumulating inside the nanovesicles and replacing water, so it could protect deformable nanovesicles from disruption and achieve an acceptable encapsulated drug retention. Furthermore, trehalose is a non-reducing sugar that exists only in closed ring form, so trehalose is more flexible to form hydrogen bonds. Under such circumstances, trehalose can more easily interact with deformable nanovesicles [39], so trehalose could form a compact coating around deformable nanovesicles, resulting in a more efficient stabilization in controlling particle size and maintaining high entrapment efficiency. Therefore, in terms of maintaining the microstructure of deformable nanovesicles, trehalose is an excellent cryoprotectant. However, when trehalose is used alone, its structure is easily damaged due to the “micro-collapse” of trehalose during long-term storage [40], which can be seen from the increased PDI, the aggregation of nanovesicles and their fuzzy whorls. This type of aggregation tends to cause flocculation at first, and then preventing the nanovesicles from re-dispersing into their original distribution, and fusing into large particles during long term storage.

Lactose is a reducing sugar and can exist in both chain and ring forms [41,42]. Its chain form easily forms internal hydrogen bonds, so fewer groups of lactose are available to form hydrogen bonds with deformable nanovesicles (or phospholipids). These weak interactions between lactose and phospholipids cannot protect against the structural disruption that occurs during phase transition, so the complex may struggle to re-encapsulating nanovesicles during the freeze-drying and reconstitution processes. Therefore, the entrapment efficiency of deformable nanovesicles tends to decrease. The reducing groups of lactose can react with the amine groups of insulin to form an N-substituted glycosylamine, which is a cascade of reactions (Maillard reaction) [43], so their direct contact with insulin should be prevented. However, lactose can form a strong physical matrix that provides a physical barrier to inter nanovesicle contact, potentially related to its chain form which tends to be more rigid. This would prevent nanovesicles from fusion and aggregation. Therefore, lactose may have good cake-supporting properties to reduce mechanical damage on deformable nanovesicles, which explains why lactose can help IPC-DNVs maintain their long-term stability.

Under such circumstances, the key solution to offset the damage caused by micro-collapse is to increase the rigidity of the supporting structure, which can be achieved when trehalose is combined with lactose. The combination can effectively prevent the increased particle size and the Maillard reaction caused by lactose. When combined, lactose and trehalose have an excellent synergistic effect. Samples prepared in 8%-L-T had smaller nanovesicles with a whorl-like structure, smooth surfaces, and strong structures. The combination also achieved high entrapment efficiency and deformability index and produced long-term stability. 

For lyophilized samples, monitoring of crystallinity was important. From the XRD results, we found that the integrity of the insulin-phospholipid complex (IPC) was well protected. No crystal formed after freeze-drying. In contrast, samples were all in the amorphous state, which might be the main reason why IPC was in a good state. Crystals can damage IPC through mechanical stresses, causing the insulin to drop out, it could also result in loss of close contact between sugar and phospholipid so the IPC cannot be well fixed on the nanovesicles. Hence, crystallization needs to be avoided during freeze-drying. Another vital indicator is *T*_g_, which can be used for the selection of the best storage temperature. The storage temperature should be maintained below the *T*_g_ of the samples. From the DSC result, 8%-L-T had the highest *T*_g_ among the three, which might contribute to the high *T*_g_ of trehalose.

More importantly, this combination may also protect the integrity of the encapsulated drug, especially drugs with a high polarity like insulin, which exists between phospholipid bilayers. The CD spectra established the impact of freeze-drying on protein stability. We found 8%-T has a relatively good protective effect that may be related to the function of trehalose. Trehalose can act as a substitute for water molecules via the formation of hydrogen bonds with deformable nanovesicles [44]. Also, the chains of trehalose have good flexibilities with small influence by steric hindrance, which contribute great capabilities in stabilizing insulin. We found 8%-L is able to interact with the insulin surface but leave gaps open due to its rigid support structure. Simultaneously, the rigid part of lactose could stress the structure of insulin, so it tightens the structure of insulin as its α-helix ratio and θ208/θ223 ratio of insulin increase. In that sense, the physical instability of insulin may result from its partial folding. For better understanding their internal mechanism, we tested the CD spectra (8%-T and 8%-L-T) after six months’ storage. Insulin in the presence of flexible trehalose (8%-T) was less stable than when a combination of rigid lactose and flexible trehalose (8%-L-T) was used because the conformational stability of the insulin was affected by the collapse of trehalose during long-term storage. We speculate that lactose can form a rigid support structure by its chain form so that trehalose can persistently maintain its effect. Therefore, the combination of lactose and trehalose could preserve the native structure of insulin that exists between phospholipid bilayers in the long term. 

The most important function of proteins is their biological activity, we have examined the metabolism of insulin and 8%-L-T in isolated aminopeptidase N. After 3 h of incubation with aminopeptidase N at a concentration of 0.625 μg/mL, the remaining percentage of insulin in insulin solution and 8%-L-T was 95.86% and 98.84% (n = 3), respectively. The results indicated that insulin alone that is stable when entrapped in the complex which would be stable for buccal administration (The detail of this experiment can be found in Supplementary Materials). Besides, in vivo studies were used to investigate the effect of lyophilization. Firstly, when 8%-L-T was subcutaneously administered, 8%-L-T-SC had the same hypoglycemic properties as insulin, which meant insulin could be quickly released from the nanovesicles. This also demonstrated that the bioactivity of insulin entrapped in 8%-L-T was well maintained. Although buccal administration of 8%-L-T delayed the hypoglycemic effect compared with subcutaneous administration of 8%-L-T, the detention might due to mucosal absorption of deformable nanovesicles rather than the release of insulin. When the nanovesicles reached the blood circulation system, a significant drop in the blood glucose level was achieved. Besides, the relative bioavailability of 8%-L-T is almost the same as their relative pharmacological bioavailability, indicating that only the insulin released from the nanovesicles can work. The experimental results further confirm that insulin can be released quickly after the deformable nanovesicles entering the blood. Secondly, since 8%-L-T had the same characteristics as IPC-DNVs apart from their slightly increased size, the different hypoglycemic properties after mucosa administration might be attributed to their slightly increased size. We assumed that within the deformable ability of deformable nanovesicles, the hypoglycemic ability was well maintained. However, larger-sized deformable nanovesicles can result in higher mucosal deposition, which mainly function as drug reservoirs, especially in this system using insulin–phospholipid complex. The form of complex greatly increases the lipophilic nature of the insulin, thereby producing a much more stable hypoglycemic effect. This phenomenon slows the absorption of insulin, causing a slight delay in the hypoglycemic effect. If the size of deformable nanovesicles increases to 10 times that of the limited gap of the upper layer of mucosa, deformable nanovesicles will mainly work as conventional nanovesicles and they may struggle to pass through the upper layer of mucosa via deformation. Under such circumstances, deformable nanovesicles mainly display a penetration enhancing effect, and nanovesicles fuse with the surface of mucosa to pass through the mucosa.

**Figure 10:**
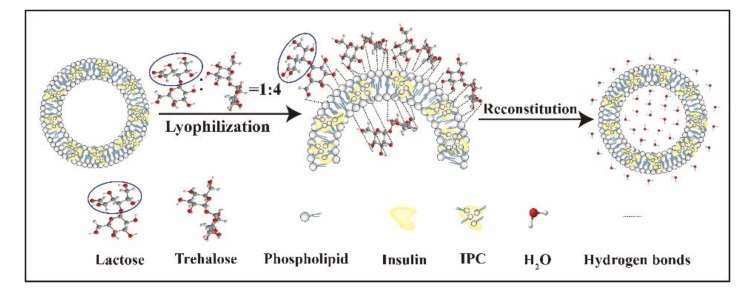
Protection mechanisms of the combination using of trehalose and lactose.

Because a combination of lactose and trehalose was used, IPC-DNVs in proper density could not only control their size, PDI, and maintain their entrapment efficiency, deformability index, and micro-structure after freeze-drying, but also maintain their long-term stability and bioactivity. The possible protection mechanisms of 8%-L-T are shown in Figure 10.

## 5. Conclusions

We successfully fabricated stable lyophilized deformable nanovesicles based on an insulin-phospholipid complex (IPC-DNVs) for buccal (mucosa) administration by combining lactose and trehalose. Based on our experimental findings above, we found that diluted samples lyophilized with 8% lactose and trehalose at a ratio of 1:4 (8%-L-T) efficiently produced stable IPC-DNVs with good long-term stability, which completely meets the stringent production requirements. We suggest the following mechanisms to explain the protection behavior of the combination cryoprotectants. Trehalose can maintain the low molecular mobility of individual vesicles and reduce the surface tension of the vesicles during freeze-drying so that smaller nanovesicles form, the dehydration stress between the inside and outside of the nanovesicles can be offset by the accumulation of trehalose inside the nanovesicles during freeze-drying, controlling particle size and maintaining good entrapment efficiency (EE, %) as well as a high deformability index (DI). Simultaneously, lactose can form a physical protective matrix that is smooth and rigid. Thus, it may help nanovesicles resist fusion, aggregation, and mechanical damage during long-term storage. Finally, compared with IPC-DNVs, the relative bioavailability *(Fr)* and relative pharmacological bioavailability *(Fp)* of the lyophilized samples were identical, the combined cryoprotectants can completely preserve the bioactivity of the protein and intrinsic structure of deformable nanovesicles. Our results provide new insight into the selection of density of nanovesicles, reconstituted solvent, and cryoprotectants. Next, we are focusing on a deeper investigation of the fabrication of stable deformable nanovesicles through vacuum drying or spray drying, which require almost the same nanovesicle density, type, ratio, as well as amount of cryoprotectants. 

## Figures and Tables

**Table 1:** Effect of different density of nanovesicles on the size, PDI, entrapment efficiency, and deformability index of IPC-DNVs after lyophilization (*n* = 3).

Formulation		Density (per μm^3^)	Size (nm)	PDI	EE (%)	DI (µg/cm^2^/s)
**Dilution**	0	1000	149.87 ± 0.70	0.49 ± 0.00	80.92 ± 1.84	33.20 ± 0.21
	1	500	109.23 ± 0.76	0.20 ± 0.09	80.50 ± 0.36	36.31 ± 2.44
	3	333	94.45 ± 3.11	0.28 ± 0.02	79.06 ± 0.20	37.13 ± 1.48
	5	200	85.37 ± 0.29	0.25 ± 0.01	60.69 ± 1.06	40.31 ± 2.81

Lyophilized IPC-DNVs using 8% trehalose as a cryoprotectant.

**Table 2:** Effect of different reconstituted solvent on the size, PDI, entrapment efficiency, and deformability index of IPC-DNVs after lyophilization (*n* = 3).

Reconstituted Solvent	Size (nm)	PDI	EE (%)	DI (µg/cm^2^/s)
Phosphate buffer	131.57 ± 1.62	0.21 ± 0.11	81.18 ± 0.52	31.04 ± 0.12
Acetate buffer	139.60 ± 0.56	0.26 ± 0.01	73.68 ± 0.49	33.92 ± 0.51
Sodium chloride	122.93 ± 0.61	0.21 ± 0.01	75.90 ± 0.49	36.51 ± 1.49
Water	105.57 ± 0.59	0.20 ± 0.00	83.37 ± 0.18	36.91 ± 1.41

Lyophilized IPC-DNVs using 8% trehalose as cryoprotectants.

**Table 3:** Effect of different cryoprotectants on the appearance, size, PDI, entrapment efficiency, and deformability index of IPC-DNVs after lyophilization (*n* = 3).

Cryoprot-ectant ^a^	Reconstituted after Lyophilization						Storage at 25 °C for 1 month					
	Lyo ^b^	Rec ^c^	Size(nm)	PDI	EE (%)	DI (µg/cm^2^/s)	Lyo ^b^	Rec ^c^	Size(nm)	PDI	EE (%)	DI (µg/cm^2^/s)
Glucose	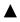	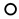	279.77 ± 2.31	0.45 ± 0.00	73.16 ± 1.91	23.09 ± 3.54	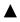	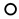	335.10 ± 3.62	0.62 ± 0.13	67.42 ± 2.11	22.15 ± 3.79
Sucrose	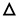	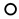	181.83 ± 0.25	0.28 ± 0.01	71.19 ± 5.26	32.02 ± 4.29	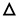	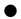	237.30 ± 1.15	0.34 ± 0.00	68.86 ± 2.74	27.91 ± 3.46
Lactose	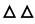	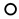	151.03 ± 0.46	0.16 ± 0.01	70.19 ± 2.13	34.03 ± 3.72	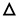	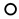	155.07 ± 0.31	0.16 ± 0.01	70.02 ± 0.69	34.21 ± 4.80
Trehalose	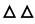	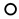	105.57 ± 0.59	0.22 ± 0.00	80.68 ± 1.05	37.62 ± 3.18	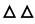	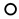	128.23 ± 2.95	0.32 ± 0.01	84.62 ± 0.18	34.29 ± 2.52
Mannitol	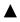	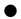	450.70 ± 5.99	0.37 ± 0.13	64.31 ± 9.88	22.05 ± 1.39	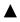	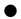	627.97 ± 5.62	0.43 ± 0.03	24.45 ± 9.17	21.01 ± 1.86
Inulin	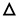	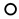	385.53 ± 9.32	0.31 ± 0.19	73.53 ± 4.29	14.59 ± 4.50	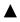	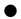	409.44 ± 1.49	0.31 ± 0.08	73.01 ± 3.24	10.39 ± 2.85
Glycine	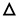	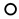	249.28 ± 6.20	0.40 ± 0.12	64.20 ± 3.19	29.94 ± 2.05	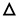	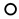	269.77 ± 3.40	0.41 ± 0.06	63.05 ± 5.12	27.45 ± 3.07
Hyaluronan	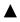	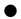	835.87 ± 50.00	0.60 ± 0.09	80.32 ± 1.07	8.39 ± 1.94	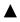	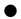	-	-	82.33 ± 0.46	3.21 ± 0.30

a: the concentration of cryoprotectants was fixed at 8%, b: lyophilized appearance, 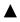 non-caked, critically shrunken, sprayed, or loose, 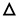 slightly shrunken, 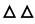 caked and compact, c: reconstituted appearance, 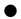 aggregated, 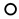 translucent. “-” means the data could not be obtained because the reconstituted samples were aggregated.

**Table 4:** Effect of different ratios and amount of lactose and trehalose on the size, PDI, entrapment efficiency and deformability index of IPC-DNVs after lyophilization (*n* = 3).

Ratio (*w*/*w*)	Size (nm)	PDI	EE (%)	DI (µg/cm^2^/s)		Size (nm)	PDI	EE (%)	DI (µg/cm^2^/s)
	Reconstituted after Lyophilization								
1:2	131.67 ± 0.42	0.20 ± 0.02	73.76 ± 0.42	35.42 ± 2.52	3%	149.27 ± 2.01	0.49 ± 0.00	59.26 ± 2.49	37.21 ± 3.07
1:4	109.47 ± 0.49	0.18 ± 0.01	80.80 ± 1.48	38.47 ± 4.13	5%	122.33 ± 1.03	0.28 ± 0.01	77.47 ± 1.88	37.91 ± 4.18
1:6	111.00 ± 0.72	0.22 ± 0.01	80.82 ± 0.97	37.95 ± 3.45	8%	109.47 ± 0.49	0.18 ± 0.01	80.80 ± 1.48	38.47 ± 4.13
1:8	111.60 ± 0.46	0.26 ± 0.00	80.79 ± 0.78	37.85 ± 2.57	10%	150.53 ± 1.66	0.45 ± 0.01	80.68 ± 1.05	32.94 ± 1.09
					13%	175.00 ± 1.13	0.28 ± 0.22	83.77 ± 1.60	32.00 ± 2.49
	Storage at 25°C for 1 month								
1:2	149.00 ± 0.99	0.48 ± 0.01	71.47 ± 1.23	32.95 ± 3.25	3%	170.80 ± 0.46	0.43 ± 0.01	51.71 ± 3.45	28.39 ± 4.80
1:4	110.10 ± 0.78	0.17 ± 0.01	82.24 ± 3.65	37.59 ± 2.02	5%	130.57 ± 0.80	0.29 ± 0.12	83.12 ± 1.68	37.48 ± 3.32
1:6	114.80 ± 0.30	0.29 ± 0.02	80.64 ± 2.29	35.73 ± 3.08	8%	110.10 ± 0.78	0.22 ± 0.01	82.24 ± 0.36	37.59 ± 2.02
1:8	123.17 ± 0.45	0.27 ± 0.00	78.14 ± 0.81	33.48 ± 4.19	10%	159.00 ± 0.27	0.16 ± 0.02	81.73 ± 1.31	32.52 ± 2.91
					13%	174.43 ± 2.29	0.27 ± 0.01	80.74 ± 2.12	30.31 ± 1.14
	Storage at 25°C for 2 months								
1:2	156.17 ± 0.61	0.15 ± 0.01	70.84 ± 2.70	25.01 ± 2.75	3%	198.70 ± 2.00	0.45 ± 0.02	41.26 ± 5.80	20.52 ± 2.45
1:4	119.77 ± 0.45	0.18 ± 0.02	81.50 ± 7.97	37.02 ± 4.50	5%	141.10 ± 1.22	0.33 ± 0.07	80.28 ± 5.18	38.26 ± 2.71
1:6	119.37 ± 0.25	0.33 ± 0.01	80.25 ± 2.63	34.42 ± 4.50	8%	119.77 ± 0.45	0.21 ± 0.01	81.50 ± 7.97	37.02 ± 4.50
1:8	176.70 ± 0.72	0.58 ± 0.02	74.32 ± 2.77	29.84 ± 4.45	10%	168.47 ± 0.55	0.43 ± 0.01	84.55 ± 2.17	28.75 ± 3.41
					13%	181.8 ± 1.14	0.47 ± 0.01	85.20 ± 1.57	30.16 ± 2.95

**Table 5:** *T*_g_ values of lyophilized samples.

Formulation	*T*_g_ values(°C)
8%-T	57.12
8%-L	38.09
8%-L-T	50.42

**Table 6:** α-helix and θ208/θ223 ratios of lyophilized IPC-DNVs.

Formulation	α-helix Ratio (%)	θ208/θ223 Ratio
Insulin	23.5	1.62
IPC-DNVs	23.3	1.63
8%-L	35.9	2.30
8%-T	24.1	1.42
8%-L-T	23.8	1.61

**Table 7:** The relative bioavailability (*Fr*) and relative pharmacological bioavailability (*Fp*) of 8%-L-T.

Formulation	AUC_0__→6h_ (mU/L·h)	*F_r_* (%)	AAC_0__→6h_ (%·h)	*F_p_* (%)
Insulin solution (1 IU/kg, SC)	239.45 ± 45.08	/	163.42 ± 30.21	/
IPC-DNVs (10 IU/kg, buccally)	437.44 ± 68.55*	18.27	247.22 ± 4.82**	15.74
8%-L-T (10 IU/kg, buccally)	418.00 ± 51.45*	17.45	236.61 ± 37.94*	14.49
8%-L-T-SC (1 IU/kg, SC)	235.95 ± 54.49	98.54	157.89 ± 15.07	96.61

*P < 0.05 between insulin solution and IPC-DNVs, *P < 0.05 between insulin solution and 8%-L-T.

## Supplementary Materials

“Stability of Deformable Nanovesicles to Begradation by Buccal Enzymes” is available online at: https://www.mdpi.com/1999-4923/11/10/539/s1.
