# Supplementary material for: Stabilization of Deformable Nanovesicles Based on Insulin-Phospholipid Complex by Freeze-Drying
Source: Pharmaceutics. 2019 Oct 16;11(10):539. doi: 10.3390/pharmaceutics11100539 (PMC6835673; doi:10.3390/pharmaceutics11100539)
Supplement: Supplementary file 1 [file pharmaceutics-11-00539-s001.pdf]

pharmaceutics

MDPI

Supplementary Materials: Stabilization of Deformable Nanovesicles Based on Insulin-Phospholipid Complex by Freeze-Drying

You Xu, Yiyue Guo, Yuqi Yang, Yingying Meng, Xuejue Xia and Yuling Liu

Stability of Deformable Nanovesicles to Begradation by Buccal Enzymes

Method

The stability of insulin and the lyophilized IPC-DNVs to isolated aminopeptidase N was evaluated by incubating the samples with artificial saliva, which contains isolated aminopeptidase N. Briefly, 100 μL of reconstituted IPC-DNVs suspension was diluted with artificial saliva containing aminopeptidase N (0.625 μg/mL) to 10 mL and incubated in a shaking water bath at 37°C and 100 rpm. At different time intervals, a 500 μL aliquot was withdrawn and placed into an ice bath for 2 min to stop the reaction. Thereafter, the samples were measured using the RP-HPLC assay mentioned in Section 2.5. Finally, the integrity of deformable nanovesicles were analyzed by testing the particle size, PDI, entrapment efficiency and deformability index. Each experiment was performed in triplicate.

Result and discussion

In this study, we examined the metabolism of insulin and 8%-L-T of isolated aminopeptidase N The metabolism of insulin was monitored by measuring the decrease in the peak area of the native insulin over time. After 3 h of incubation with aminopeptidase N at a concentration of 0.625 μg/mL, the remaining percentage of insulin in insulin solution and 8%-L-T was 95.86% and 98.84% (n = 3), respectively. The degree of degradation in the two groups was not significantly different.

After incubation with buccal enzymes for 3 h, the mean diameter of 8%-L-T was 105.38± 3.52 nm with the same distribution as before. The entrapment efficiency and deformability index were 80.28% ± 1.77% and 37.05% ± 3.13 g/cm^2/s (n=3), respectively.

The activity of enzymes in the buccal cavity is relatively low compared with the activity of digestive enzymes. Buccal administration of some peptide drugs is limited due to drug degradation by membrane-bound peptidases. Aminopeptidase N is the most abundant peptidase in the buccal mucosa and the enzymes are present on the surface of the buccal mucosa [1]. In this study, isolated aminopeptidase N was used to evaluate the stability of insulin and 8%-L-T in the buccal cavity by determining whether native insulin and 8%-L-T were stable in artificial saliva containing aminopeptidase. The results indicated that insulin alone that is stable when entrapped in the complex would be stable for buccal administration. More importantly, the structure of deformable nanovesicles was still retained, providing support for the buccal administration of insulin.

References

1. Langoth N, Bernkop-Schnurch A, P K. The inhibitory effect of glutathione on buccal enzymatic degradation of therapeutic peptides (leu-enkephalin, luteinizing hormone-releasing hormone and pituitary adenylate cyclase activating peptide). *J Durg Deliv Sci Tec.* **2005**;*15*:435-438.
